# Transcriptomic and network analyses reveal distinct nitrate responses in light and dark in rice leaves (*Oryza sativa* Indica var. Panvel1)

**DOI:** 10.1038/s41598-020-68917-z

**Published:** 2020-07-22

**Authors:** Ravi Ramesh Pathak, Annie Prasanna Jangam, Aakansha Malik, Narendra Sharma, Dinesh Kumar Jaiswal, Nandula Raghuram

**Affiliations:** University School of Biotechnology, Guru Gobind Singh Indraprastha University, Sector 16C, Dwarka, New Delhi 110078 India

**Keywords:** Plant molecular biology, Plant signalling

## Abstract

Nitrate (N) response is modulated by light, but not understood from a genome-wide perspective. Comparative transcriptomic analyses of nitrate response in light-grown and etiolated rice leaves revealed 303 and 249 differentially expressed genes (DEGs) respectively. A majority of them were exclusive to light (270) or dark (216) condition, whereas 33 DEGs were common. The latter may constitute response to N signaling regardless of light. Functional annotation and pathway enrichment analyses of the DEGs showed that nitrate primarily modulates conserved N signaling and metabolism in light, whereas oxidation–reduction processes, pentose-phosphate shunt, starch-, sucrose- and glycerolipid-metabolisms in the dark. Differential N-regulation of these pathways by light could be attributed to the involvement of distinctive sets of transporters, transcription factors, enriched cis-acting motifs in the promoters of DEGs as well as differential modulation of N-responsive transcriptional regulatory networks in light and dark. Sub-clustering of DEGs-associated protein–protein interaction network constructed using experimentally validated interactors revealed that nitrate regulates a molecular complex consisting of nitrite reductase, ferredoxin-NADP reductase and ferredoxin. This complex is associated with flowering time, revealing a meeting point for N-regulation of N-response and N-use efficiency. Together, our results provide novel insights into distinct pathways of N-signaling in light and dark conditions.

## Introduction

A major challenge in improving crops for input use efficiency is to understand and optimize the inputs for various agroclimatic conditions including light and photoperiod, soil type, altitude, humidity etc. Nitrogen (N) is quantitatively the most important fertilizer input for intensive cropping, but globally, nitrogen use efficiency (NUE) is as low as 30–40% for various crops, which is a major cause for economic losses and environmental consequences of N pollution^1^. Rice has the least NUE among cereals and therefore tops all other crops in N-fertilizer consumption in India^2^. The molecular aspects of nitrate transport, assimilation, signalling and crosstalk with water, hormone, and development are better understood than the biological determinants of crop nitrogen use efficiency^3–14^. Characterization of the phenotype for NUE will be crucial for progress in this regard^15^.

Nitrate is taken up into the cell by a family of transporters and converted into ammonium ions by the serial action of nitrate reductase (*NR*) and nitrite reductase (*NiR*), followed by their assimilation into amino acids through the glutamine synthetase and glutamate synthase (GS-GOGAT) cycle. This requires 2-oxoglutarate (2-OG) from the carbon metabolism and hence coordination between C and N metabolism^4^. Transcriptomic studies have revealed thousands of nitrate-responsive genes in Arabidopsis^3,16–18^, rice^19–23^ and maize^24^. They include those involved in metabolism, redox balance, signaling, stress, hormones, development etc., indicating their possible role in NUE^6,8,10,25,26^. Heterotrimeric G-protein gamma subunit has been identified as a QTL for NUE in rice^27^, while the beta subunit has been shown to mediate nitrate-responsive root development^28^. G-proteins were implicated in light regulation of NR gene expression in our earlier studies in maize^29,30^ and rice^31^. Our transcriptome studies in G-protein mutants showed their role in other nitrogen and/or stress responses^32–36^.

Light is an important regulator of plant N-responses, both through photosynthesis and C/N balance, as well as through light signaling^37^. Light promotes N uptake and/or assimilation in maize^29^, rice^38^ and Arabidopsis^39,40^. Recently, elongated hypocotyl 5 (*Hy5*), a positive regulator of light signalling, has been shown to enhance nitrate uptake in Arabidopsis^40^. Interestingly, N regulates flowering time by modulating a blue-light receptor cryptochrome 1 (*CRY1*) in Arabidopsis^41^. Sucrose mimics light-responses and its exogenous application induces NR activity possibly via hexose’s sensor independent N-signalling pathway in Arabidopsis^42^. Further, metabolite sensor *SNRK1* regulates NR and sucrose phosphate synthase activity and therefore controls both N and C metabolism^43^.

Most of the above information on the role of light in N-response was based on Arabidopsis and not on crop plants. Therefore, to delineate the molecular basis of light-dependent and independent nitrate response, we analysed the nitrate-responsive leaf transcriptomes of light-grown and etiolated rice seedlings in this study.

## Results

### Delineation of nitrate responses in etiolated and light-grown rice seedlings

**Figure 1:**
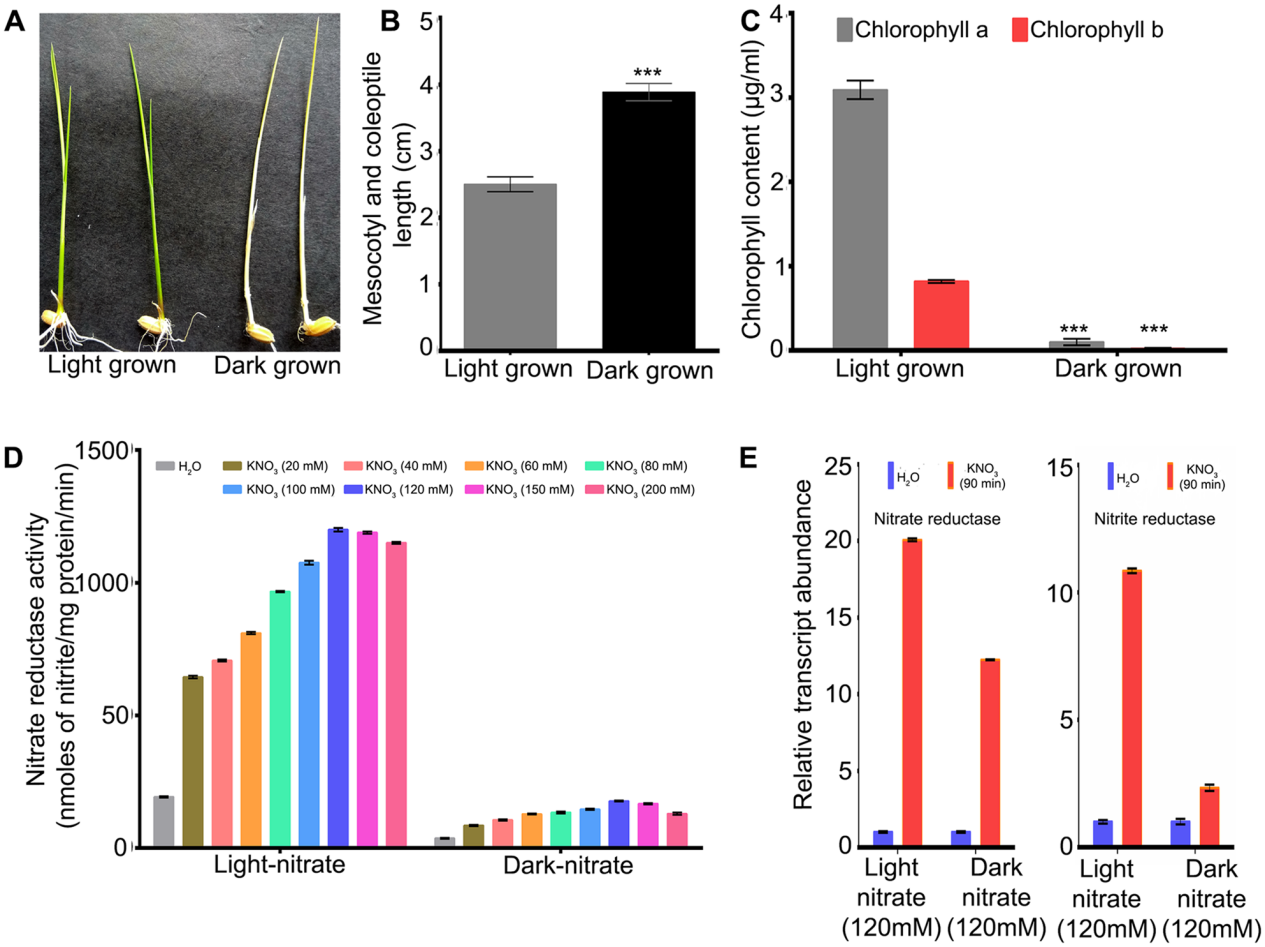
Phenotypic, physiological and molecular analyses of nitrate responses in 10 days old light-grown and etiolated seedlings in rice. (**A**) Representative image of light-grown and etiolated seedlings. (**B**) Mesocotyl and coleoptile length of light-grown and etiolated seedlings and data represent the average of 16 seedlings. (**C**) Leaf chlorophyll content. The data represents the means ± SD of three independent biological replicates. (**D**) Effect of nitrate doses on nitrate reductase activity in the leaves of light-grown and etiolated seedlings. (**E**) Relative expression level of nitrate reductase and nitrite reductase genes in the leaves of light-grown and etiolated seedlings treated with either water (control) or 120 mM nitrate for 90 min, determined by qRT-PCR. ***p value < 0.001.

The experimental conditions for analysis of nitrate response in excised leaves of etiolated and light-grown 10-days old rice seedlings were confirmed as follows. Etiolated seedlings were pale yellow in colour and showed significantly (p < 0.05) elongated mesocotyl and coleoptile length compared to light-grown condition (Fig. 1A,B). The leaf chlorophyll contents (chl-a and chl-b) were relatively higher in light-grown seedlings, as expected (Fig. 1C). The optimum concentration for nitrate treatment of excised leaves was determined by a dose–response analysis of NR activity and 120 mM KNO_3_ was found to be optimum in light and dark conditions (Fig. 1D). The NR activity was much higher in light as compared to dark condition at all doses of nitrate (Fig. 1D). This was also true for transcript levels of *NR* and *NiR* at the optimum nitrate dose (Fig. 1E). These results confirmed the conditions of etiolation and nitrate treatments for microarray analysis.

### Nitrate-responsive leaf transcriptomes in light and dark

**Figure 2:**
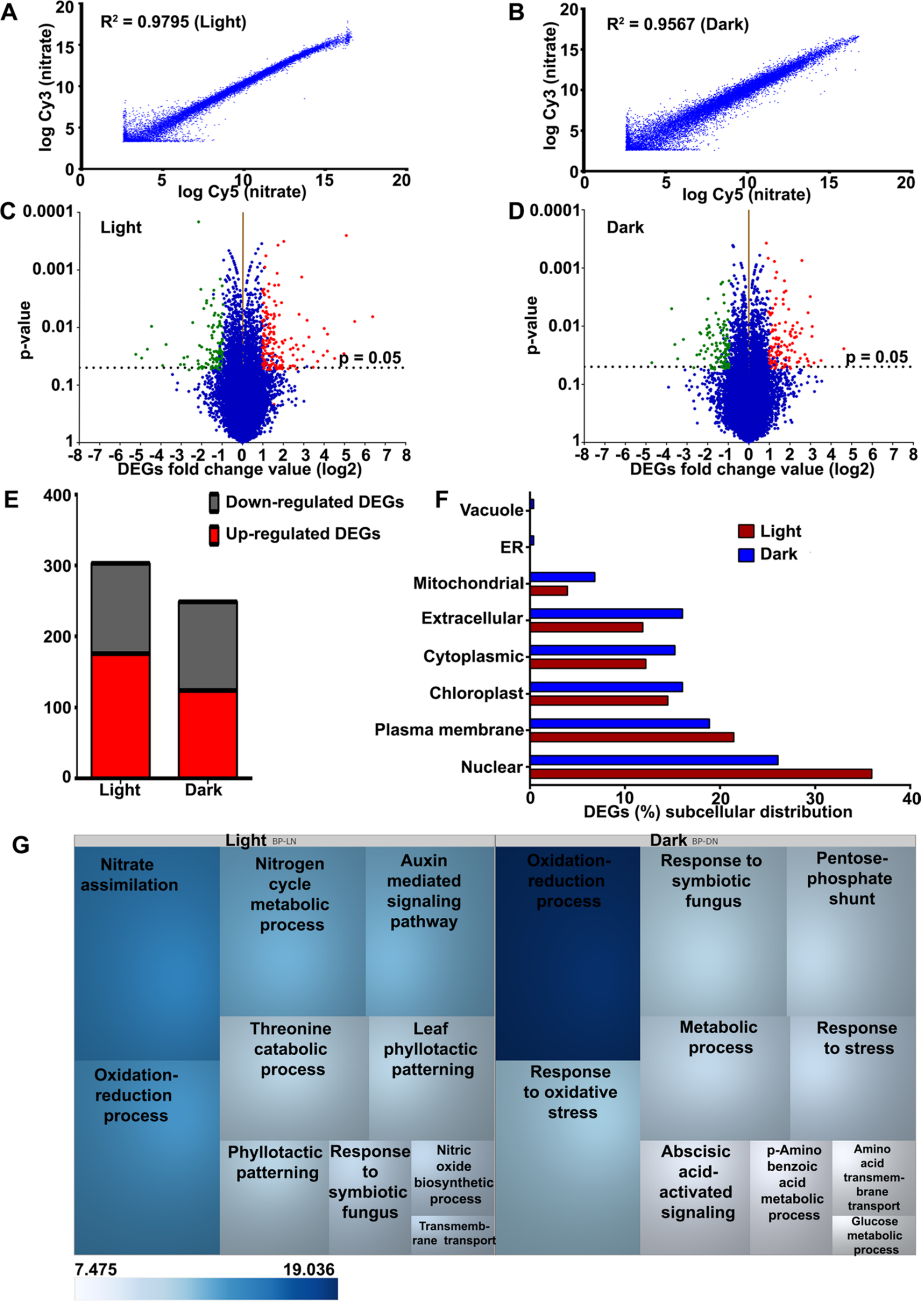
Analysis of nitrate-responsive leaf transcriptomes of light-grown and etiolated rice seedlings. Scatter plots show the correlation between the biological replicates of transcriptomes in light (**A**) and dark (**B**) conditions. The correlation coefficients of normalized ratios were calculated between the dye swapped nitrated treated samples in light (R^2^ = 0.9795) and dark (R^2^ = 0.9567). The volcano plots of microarray data are shown for light (**C**) and dark (**D**). The X-axis represents the fold change (log_2_) and p-value on Y-axis. The dashed horizontal line shown on the plots represents the p value cut-off (p = 0.05) and the genes above this line are statistically significant. Each transcript is represented by scattered dots. The red and green coloured scattered dots represent the up- and down-regulated DEGs, respectively. (**E**) Column graph shows the numbers of up-regulated or down-regulated nitrate-responsive genes in light and dark conditions. (**F**) Bar graph depicting the subcellular localization of DEGs-encoded proteins identified in light and dark. (**G**) The DEGs were functionally annotated into different biological processes using Expath tool. Top 10 statistically significant biological processes (P < 0.05) were visualized using TreeMap software (https://www.treemap.com/). The size of the box is inversely proportional to the ranking of the biological processes by p value and coloured according to their statistically significant p value (−log_2_). In other words, a biological process with the lowest p value has the biggest box size and its colour intensity shows its statistical significance. *BP-LN* biological process-light nitrate, *BP-DN* biological process-dark nitrate.

Genome-wide expression profiling of nitrate-response was carried out by microarray analysis using leaves from ten days old etiolated and light-grown rice seedlings treated with or without 120 mM KNO_3_ for 90 min under light and dark conditions. Hierarchical clustering of expression values obtained for light- and dark nitrate-treated samples revealed similar trends among the two independent biological-cum-flip dye replicates (Fig. S1). Scatter plots of replicates revealed high correlations between replicates (R^2^ = 0.9795 for light-nitrate and R^2^ = 0.9561 for dark nitrate) (Fig. 2A,B), indicating data quality and reproducibility. A geomean cutoff (log_2_FC) of ± 1 and p value ≤ 0.05 were used to define the differentially expressed genes (DEGs). Multiple testing corrections using Benjamini–Hochberg adjusted p value were tried but not considered, as they produced many false negatives by eliminating many well-known N-responsive genes obtained from qualifying our geomean cutoff (log_2_FC) of ± 1 (Tables S1, S2). An overview of global gene expression using volcano plots showed that nitrate regulates substantially more DEGs in light as compared to dark condition (Fig. 2C,D). Data analysis revealed 303 nitrate-responsive DEGs in light (176 up- and 127 down-regulated) and 249 DEGs under dark condition, with 124 up- and 125 down-regulated genes (Fig. 2E). The ratio of up/down regulated genes was also higher in light, indicating substantial transcriptional reprogramming and molecular changes in light.

We have used CELLO tool (https://cello.life.nctu.edu.tw/)^44,45^ to predict the subcellular distribution of proteins encoded by nitrate-responsive DEGs in light and dark, which revealed that they are predominantly targeted to the nucleus, followed by plasma membrane, chloroplast and others (Fig. 2F). Their comparative analyses revealed that more such proteins were localized to the nucleus in light, whereas there were more extracellular proteins in the dark. The latter may be involved in etiolated phenotype in the dark, as genes encoding extracellular or cell wall proteins are known for their role in cell expansion^46^. The secretory nature and the presence of other signal peptides of proteins encoded by DEGs were analyzed using TargetP 2.0. We observed that a higher percentage of DEGs encoded proteins are non-secretory in nature in both light and dark conditions (Fig. S2).

To understand the nitrate-responsive biological processes, we functionally annotated the DEGs based on their GO enrichment analysis using ExPath tool^47^. It revealed that in addition to the expected enrichment of DEGs involved in nitrate signaling and metabolism, light-grown seedlings showed auxin-mediated signaling and leaf phyllotactic patterning, among others (Fig. 2G). However, in the dark, oxidation–reduction processes, pentose-phosphate shunt and oxidative stress response were the top-most significantly enriched GO terms for nitrate response (Fig. 2G). The details of GO-enrichment analyses are provided in Table S3.

**Figure 3:**
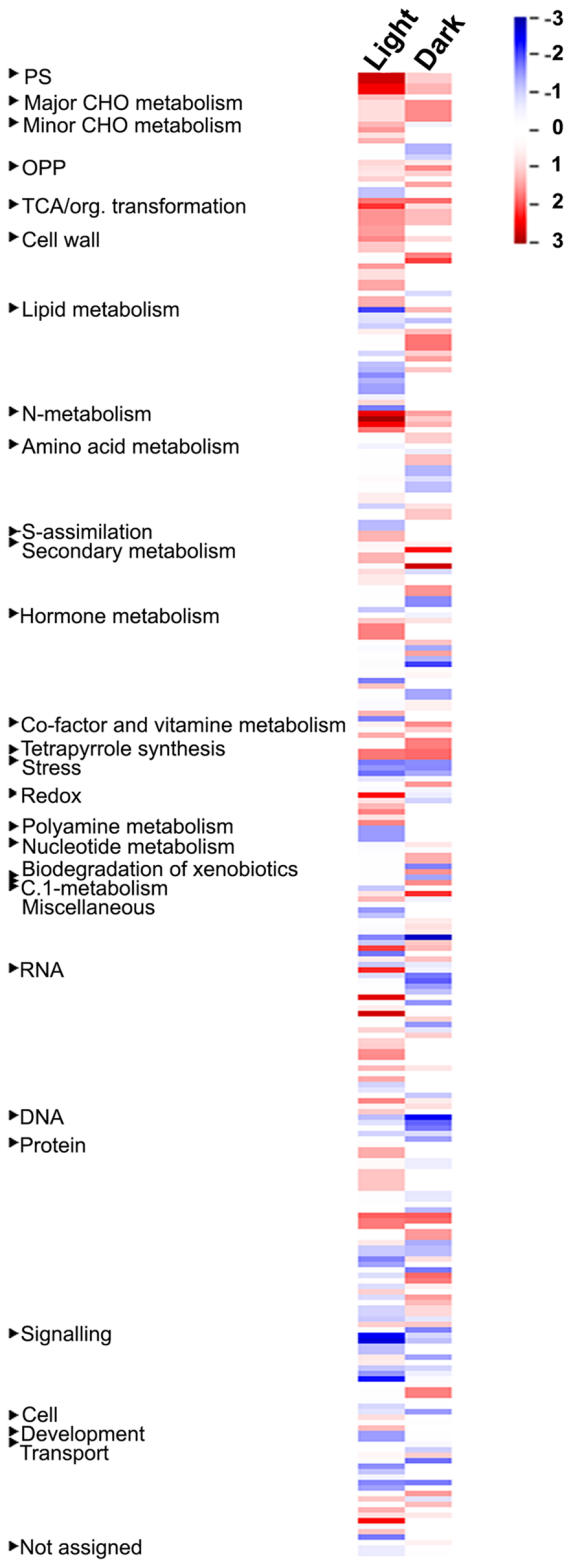
PageMan analyses of nitrate-responsive transcripts differentially expressed between light and dark conditions. Over- and under-represented nitrate-responsive pathways in light and dark conditions were analyzed in PageMan (https://mapman.gabipd.org/pageman) using Wilcoxon algorithm with default parameters. Bin names are shown to the left of the image and treatment conditions are mentioned at the top of the image. Significant functional groups are indicated by either red (over-represented) or blue (under-represented) colour according to the scale and details are provided in Fig. S3.

To understand the biological processes that constitute nitrate-response, we mapped the DEGs and their expression values onto various cellular pathways/processes using MapMan (https://mapman.gabipd.org/mapman)^48^. The results were broadly similar to those from GO analysis, with nitrate-responsive changes to metabolism, cellular responses, regulation and hormone biosynthesis among others (Tables S4, S5). Further, a number of DEGs were found to be involved in development, biotic and abiotic stresses, suggesting signaling crosstalk between nitrate and stress responses in plants^26^. PageMan analysis revealed the over- and under-representation of different pathways affected by nitrate in light and dark conditions. The significantly enriched categories were photosynthesis, nitrogen metabolism, amino acid metabolism, carbohydrate metabolism, signaling and development among others (Fig. 3).

Further, ExPath-based comparative pathway analyses revealed the enrichment of statistically significant (p value < 0.05) pathways such as phenylpropanoid biosynthesis, nitrogen metabolism, phenylalanine metabolism, hormone signal transduction and photosynthesis in light, whereas in the dark, they were starch and sucrose metabolism, metabolic pathways, glycerolipid metabolism and N-Glycan biosynthesis (Table S6).

### Validation of N-responsive genes involved in nutrients, stress and development

**Figure 4:**
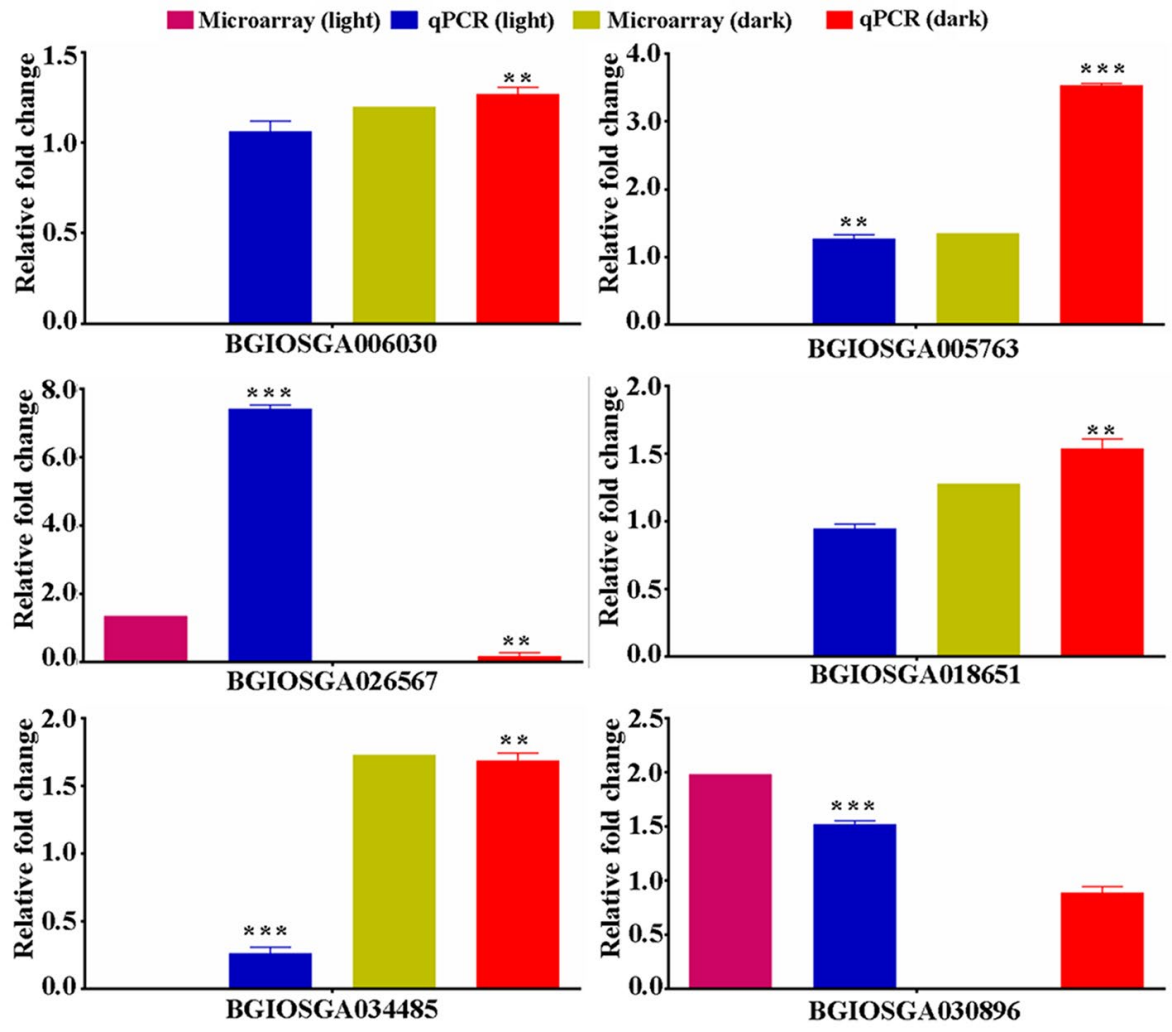
qRT-PCR validation of nitrate-responsive genes identified in light and dark conditions. Total RNA was isolated from control (H_2_O) and nitrate-treated leaves of 10 days old light-grown and etiolated rice seedlings. Relative transcript abundance was calculated by comparative Ct method using actin as a reference gene for data normalization. The data represent the mean ± SE of three technical replicates. Final relative fold change values (qPCR) of DEGs and their corresponding values obtained in microarray experiment were plotted for parallel comparison of DEG trends in qPCR and microarray experiments. To avoid additional bars and better clarity of the results, control value (H_2_O) obtained in qPCR were not included in the image. However, the statistical analyses were performed between control (H_2_O) and nitrate treated value (comparative Ct method) obtained by qPCR. Statistical unpaired T test analyses [control (H_2_O) vs. nitrate] were performed in the GraphPad Prism 6 software using technical replicates (**p value < 0.01, ***p value < 0.001). The experiments were performed with three independent biological replicates. MYB family transcription factors (BGIOSGA006030, BGIOSGA018651); universal stress protein domain containing protein (BGIOSGA005763); calcium-dependent protein kinase (BGIOSGA026567); efflux transporter of nicotianamine 1 (BGIOSGA034485); bHLH120 (BGIOSGA030896).

To validate the expression profile of nitrate-responsive genes involved in nutrient, stress and development, 6 DEGs out of 8 tested DEGs encoding transporter, transcription factors and others showed statistically significant differences in qRT-PCR, thus validating the microarray results (Fig. 4).

The details of these DEGs and corresponding primer sequences used for qRT-PCR are provided in Table S7. Three of them were up-regulated by nitrate in light but not detected in dark condition. They were, calcium-dependent protein kinase (Os08g0540400/BGIOSGA026567) and basic helix-loop-helix proteins *bHLH120* (Os09g0455300/BGIOSGA030896), *bHLH066* (Os03g0759700/BGIOSGA013600). However, universal stress domain containing protein (Os02g0707900/BGIOSGA005763), efflux transporter of nicotianamine 1 (Os11g0151500/BGIOSGA034485) and MYB family transcription factors (Os02g0618400/ BGIOSGA006030, Os05g0195700/BGIOSGA018651) were up-regulated by nitrate in the dark but not detected in light condition. The SPX domain containing protein (Os02g0202200/BGIOSGA007749) was down-regulated in both light and dark. The reason for non-detected DEGs in microarray was their statistically non-significant expressions (Table S7). The qRT-PCR results of six DEGs confirmed their similar regulation as observed in the microarray experiments, thus validating them (Fig. 4).

### Organ association of the expression of identified N-responsive genes

**Figure 5:**
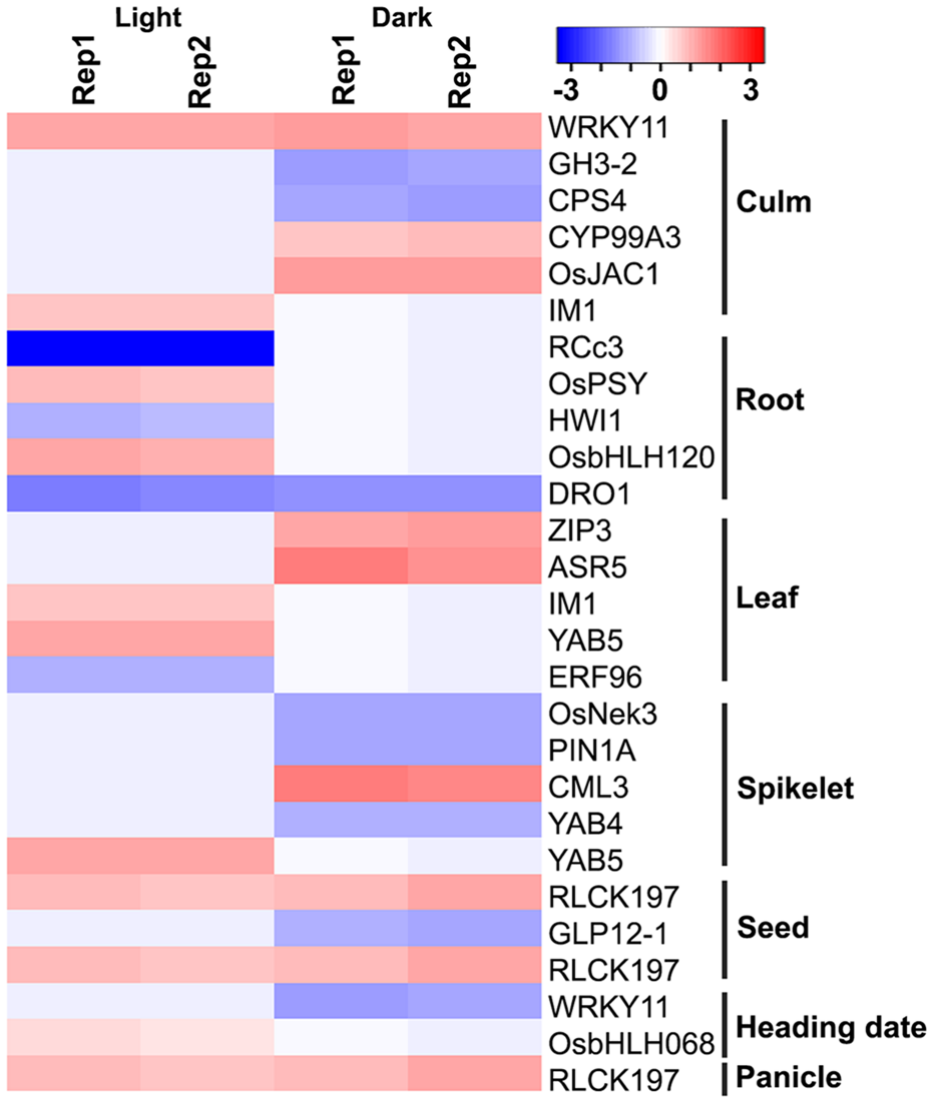
Organ-association of nitrate-responsive genes. The identified N-responsive DEGs were searched in Oryzabase database, catalogued and their expression presented as heat maps using heatmapper (https://heatmapper.ca/). The default colour scale shows the expression value of DEGs (calculated as z-scores) associated with culm, root, leaves, spikelet, seed, heading date and panicle. Rep1, replicate 1; Rep2, replicate 2. The symbols of genes were according to CGSNL.

As transcriptome analysis reveals only those genes expressed in the particular stage and tissue used (leaves of 10-days old seedlings in this case), it was of interest to examine how many of the N-responsive genes identified here are expressed in other stages/tissues of the rice plant. This allows to separate those genes that are ubiquitously expressed in most of the organs from those that are unique to specific organs/stages for further validation of their nitrate response. This was particularly relevant in view of the incomplete characterization of the phenotype for N-response/NUE. We therefore searched the *oryzabase* database (https://shigen.nig.ac.jp/rice/oryzabase/) and found that N-responsive DEGs were assigned to culm, root, leaf, spikelet, seed, heading date and panicle (Fig. 5). The availability of nitrate in the soil significantly affects the root system architecture^8,22^. Five root-associated N-responsive DEGs were identified only in light, including phytoene synthase (LOC_Os09g38320.1) and LTP family protein (LOC_Os02g44310.1). Phytoene synthase (PSY) regulates carbon flux in carotenoid biosynthesis and is required to induce carotenogenesis-dependent ABA accumulation in root under different abiotic stress conditions^49,50^. The up-regulated expression of PSY in light may be associated with root inhibition by high nitrate in rice. Leaf is the primary organ for photosynthesis in rice and application of high N dose has been shown to reduce photosynthetic NUE^51^. Among leaf-associated N-responsive DEGs, we observed up-regulated expression of Zn transporter 3 (LOC_Os04g52310.1) and ABA stress-ripening (LOC_Os11g06720.1) in light, whereas plastid terminal oxidases 1 (LOC_Os04g57320.1) and YABBY domain containing protein (LOC_Os04g45330.1) were up-reregulated in dark (Fig. 5 and Table S8).

Plastid terminal oxidase was differentially regulated during N starvation in lower organisms^52,53^, whereas YABBY domain containing proteins are known to regulate leaf morphology in plants^54^. A few DEGs were also associated with agronomically important traits (Table S8) indicating their regulation by nitrate in rice.

### Common and unique processes of N-response in dark and light

**Figure 6:**
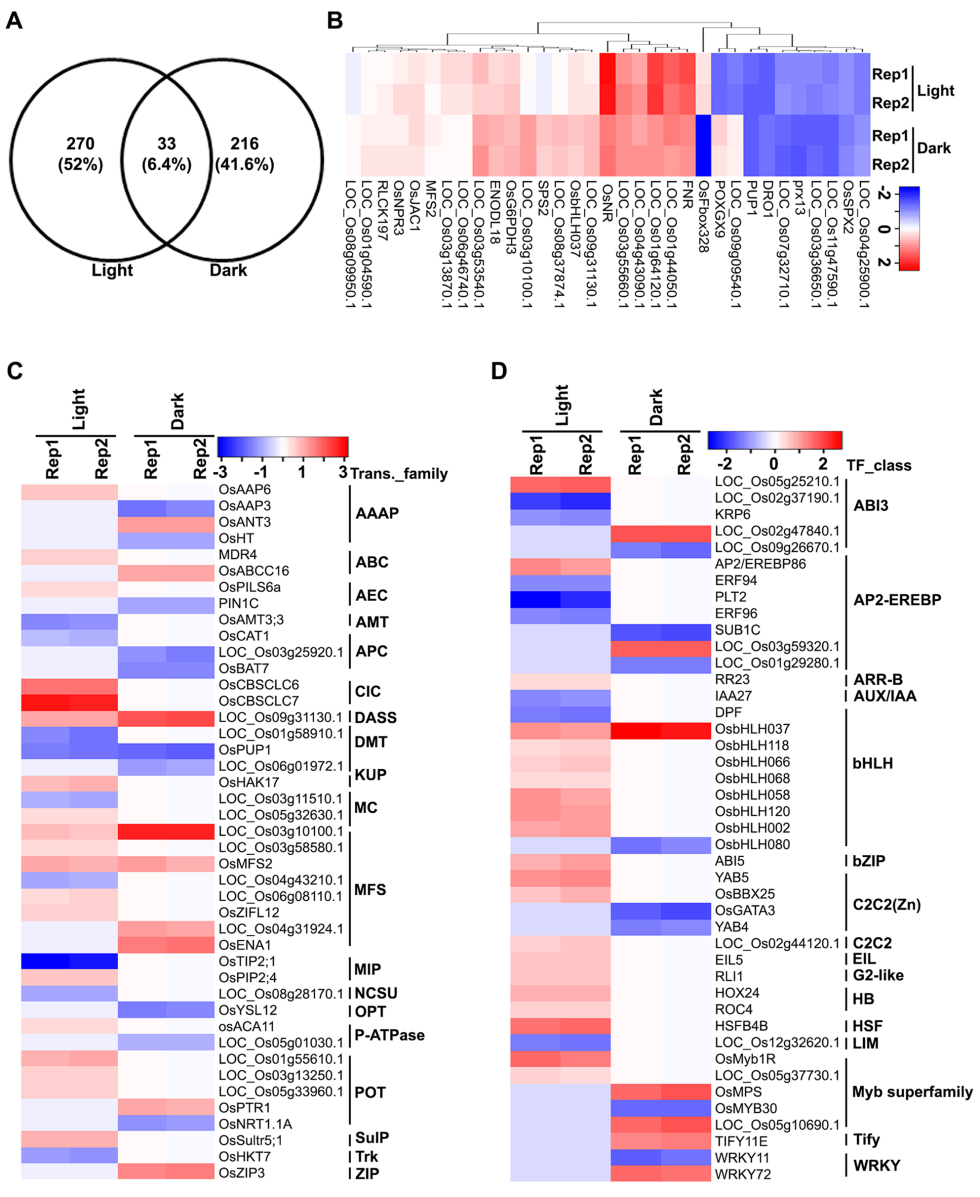
Transcriptomic analyses reveals common and distinct nitrate regulation by transporters and transcription factors in light and dark. (**A**) Venn diagram showing the number of common and exclusive DEGs identified in light and dark conditions. Heat map depicting the expression profile of common DEGs (**B**), transporters (**C**) and transcriptions factors (**D**). Each row represents individual nitrate-responsive gene and the columns correspond to light and dark conditions. The colour on scale bar matches the fold change value (log_2_FC) of DEGs calculated as z-scores. Heat maps were generated using heatmapper (https://heatmapper.ca/). *AAAP* Amino acid/auxin permease, *ABC* ATP-binding cassette, *AEC* auxin efflux carrier, *Amt* ammonia transporter channel, *APC* amino acid-polyamine-organocation, *ClC* chloride carrier/channel, *DASS* divalent anion:Na^+^ symporter, *DMT* drug/metabolite transporter, *KUP* K^+^ uptake permease, *MC* mitochondrial carrier, *MFS* major facilitator superfamily, *MIP* major intrinsic protein, *NCS2* nucleobase:cation symporter-2, *OPT* oligopeptide transporter, *P-ATPase* P-type ATPase, *POT* proton-dependent oligopeptide transporter, *SulP* sulfate permease, *Trk* K^+^ transporter, *ZIP* Zinc (Zn^2+^)-iron (Fe^2+^) permease. The symbols of genes were according to CGSNL.

To identify the similarities and differences in nitrate-response under light and dark conditions, the DEGs were subjected to venn selections using Venny 2.1.0 (https://bioinfogp.cnb.csic.es/tools/venny/), which revealed 270 and 216 N-responsive DEGs exclusive to light and dark conditions, respectively, with an additional 33 genes in common (Fig. 6A). A heat map revealed the expression pattern of common DEGs, which were associated with N-transport, metabolism, signaling and hormone (Fig. 6B) among others (Table S9).

To further segregate the common and exclusive DEGs, we performed venn selections using four different classes of DEGs i.e. up-regulated in light, down-regulated in light, up-regulated in dark and down-regulated in dark. A major fraction of DEGs were exclusive to their classes, with only 21 up-regulated DEGs common in light and dark, whereas 8 down-regulated DEGs common in light and dark (Fig. S4). MapMan analysis revealed that some genes of the ‘transport’, ‘signaling’ and associated transporter and transcription factor categories responded differently in light and dark conditions (Fig. 3).

Leaf acts as a source of N partitioning, which is mediated by phloem networks and the transport across this networks regulates N-transport pathways in root, stem and other organs. N-transport pathways are regulated by transporters and carrier proteins present in the membranous structures. Transporters are also considered as potential targets to improve NUE^55^, but it is not well known which transporters are involved in N partitioning and how N-uptake and subsequent transport towards and within different organs in plants are coordinated^56^. To catalogue different classes of N-responsive transporters known to be involved in other transport pathways, we identified 43 nitrate-responsive DEGs belonging to 19 families of transporters (Fig. 6C and Table S10) in light and dark conditions. Out of these, 9 are regulated by nitrate in both light/dark, whereas 8 are exclusive to light and 2 are unique to dark conditions only (Fig. S5). We found that nitrate up-regulated not only the expression of nitrate transporters in light, but also chloride channel protein, sulfate transporter, high-affinity potassium transporter and peptide transporter among others. Others such as sodium/sulphate symporter, sorbitol transporter and nodulin-like protein were up-regulated by nitrate in both light and dark conditions (Table S10).

To understand how nitrate regulates transcriptional networks, we identified DEGs encoding transcription factors (TFs) in rice. This was done by searching the for N-responsive DEGs in different transcription factor databases viz. RiceFREND (https://ricefrend.dna.affrc.go.jp/), RiceSRTFDB (https://www.nipgr.ac.in/RiceSRTFDB.html), STIFDB (https://caps.ncbs.res.in/stifdb/) and MapMan and classifying them into families using Rice SRTFDB (Fig. 6D and Table S11, Fig. S6). This revealed 43 TFs belonging to 16 families as regulated by nitrate in both light and dark conditions (Fig. 6D and Table S11). Among these TF families, basic helix-loop-helix (bHLH), aptela-2/ethylene-responsive element binding protein (AP2-EREBP) and MYB superfamily were the most abundant in rice. The members of bHLH, AP2-EREBP, MYB superfamily, abscisic acid-insensitive 3 (ABI3) and zinc finger C2H2 [C2C2(Zn)]TF family were regulated by nitrate in both light and dark conditions. The up-regulated TFs were predominant in light, whereas down-regulated TFs were found in both light/dark conditions (Fig. 6D, Table S11), suggesting the involvement of different TFs in mediating nitrate responses in light and dark conditions in rice.

### Different cis-acting motifs may mediate nitrate responses in light and dark

Transcription factors (TFs) act as master regulators by binding to cis-acting motifs in the promoter regions of genes to control diverse cell processes, including N signaling^9^. Identification of such motifs upstream of N-responsive genes will aid in understanding N signaling and may also reveal candidate genes for NUE. The oligo analysis program of Regulatory Sequence Analysis Tools (RSAT) was used to identify the over-represented motifs in the 1 kb promoter sequences of the N-responsive DEGs identified in light and dark conditions.

**Figure 7:**
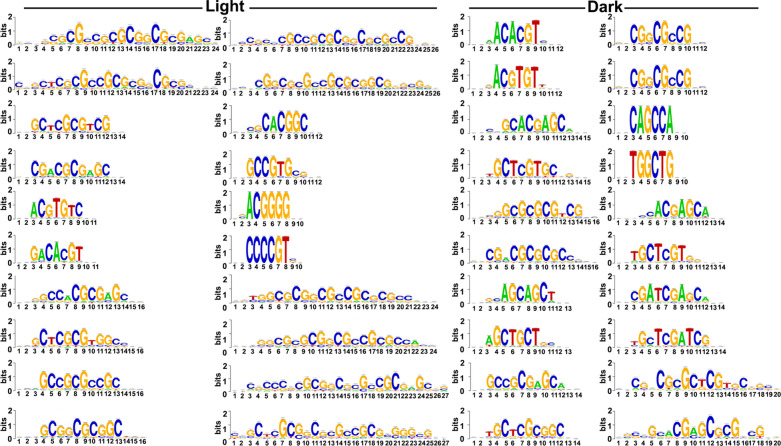
Prediction of over-represented cis-regulatory motifs in the promoter of nitrate-responsive genes. Significant enrichment of nitrate-responsive motifs in the promoter region of DEGs was identified using RSAT plantstool (https://rsat.eead.csic.es/plants/).

We separately downloaded promoter sequences of up- and down-regulated DEGs identified in light and dark conditions and predicted the enriched N-responsive motifs present in their promoter regions. Most of the predicted motifs are exclusive to their group (Tables S12, S13), which could be due to few common DEGs between light and dark (Fig. S4). A few enriched motifs predicted in the promoter of up-regulated DEGs in light condition were similar to nitrate-regulated motifs (Table 1) validated in Arabidopsis^57^. We further predicted the motif enrichment using 1 kb promoter sequence of all the DEGs identified in light and dark conditions. Examination of the 20 most significantly enriched motifs in DEGs identified under light and dark conditions revealed that they are entirely different sets, despite the fact that 33 DEGs were common to both conditions (Fig. 7 and Table S14). Many of them pertain to nitrate-regulated transcription factors known in Arabidopsis. This indicates that different TFs may mediate N-response in light and dark conditions through their respective binding motifs identified in this study.

**Table 1:** Nitrate-responsive transcription factors and their experimentally validated DNA binding sites.

TF Id (TAIR)	TF Id (RGAP)	Motif details	TF class	TF-binding-sites^Ref^	Enriched motifs in the promoter of light-nitrate up-regulated DEGs (oligo-analyses-RSAT)			
					cccgcc	Cgtggc	acgtgg	cacgtgg
At1g49720	No ortholog	ABFs binding site motif	bZIP	CACGTGGC^58^	−	+	+	+
At1g45249	LOC_Os06g10880.1	ABFs binding site motif	bZIP	CACGTGGC^58^	−	+	+	+
At4g25470	LOC_Os04g48350.1	CBF2 binding site motif	AP2-EREBP	CCACGTGG^59^	−	−	+	+
At4g36730^a^	LOC_Os06g43870.1	GBF1/2/3 BS in ADH1	bZIP	CCACGTGG^60^	−	−	+	+
At4g01120^a^	No ortholog	GBF1/2/3 BS in ADH1	bZIP	CCACGTGG^60^	−	−	+	+
At2g46270	LOC_Os01g46970.1	GBF1/2/3 BS in ADH1	bZIP	CCACGTGG^60^	−	−	+	+
At3g19290^a^	LOC_Os09g28310.1	ABRE binding site motif	bZIP	(C/T)ACGTGGC^61^	−	+	+	−
At5g65210^a^	No ortholog	TGA1 binding site motif	bZIP	TGACGTGG^62^	−	−	+	−
At2g36011	No ortholog	E2F-varient binding site motif	E2F-DP	TCTCCCGCC^63^	+	−	−	−

*Ref* reference.

^a^Nitrate regulated transcription factors in Arabidopsis^57^.

### Nitrate-responsive transcriptional regulatory networks

It is known that orthologous proteins are conserved and perform similar function in different plants. For example, nitrate transporter and other proteins involved in nitrate-associated pathways are known to perform similar function in different plants^64^. Our MapMan pathway analyses and functional classification of DEGs in light and dark conditions revealed 43 TFs as N-responsive (Fig. 6D, Table S11). Recently, nitrate-responsive transcriptional regulatory (NTR) networks have been developed in Arabidopsis^57^. Using Arabidopsis nitrate-responsive transcriptional regulatory (NTR) networks built from yeast one-hybrid screens and validated by knockouts^57^, similar networks were developed in this study using their orthologues in rice. Prior to this, we verified the level of gene conservation between rice and Arabidopsis using Orthovenn 2^65^. We observed 11,367 and 11,956 orthologous clusters in Arabidopsis and rice, respectively, of which 9,698 orthologous clusters were common (Fig. S7), confirming their high level of conservation of orthologs and associated pathways/processes.

**Figure 8:**
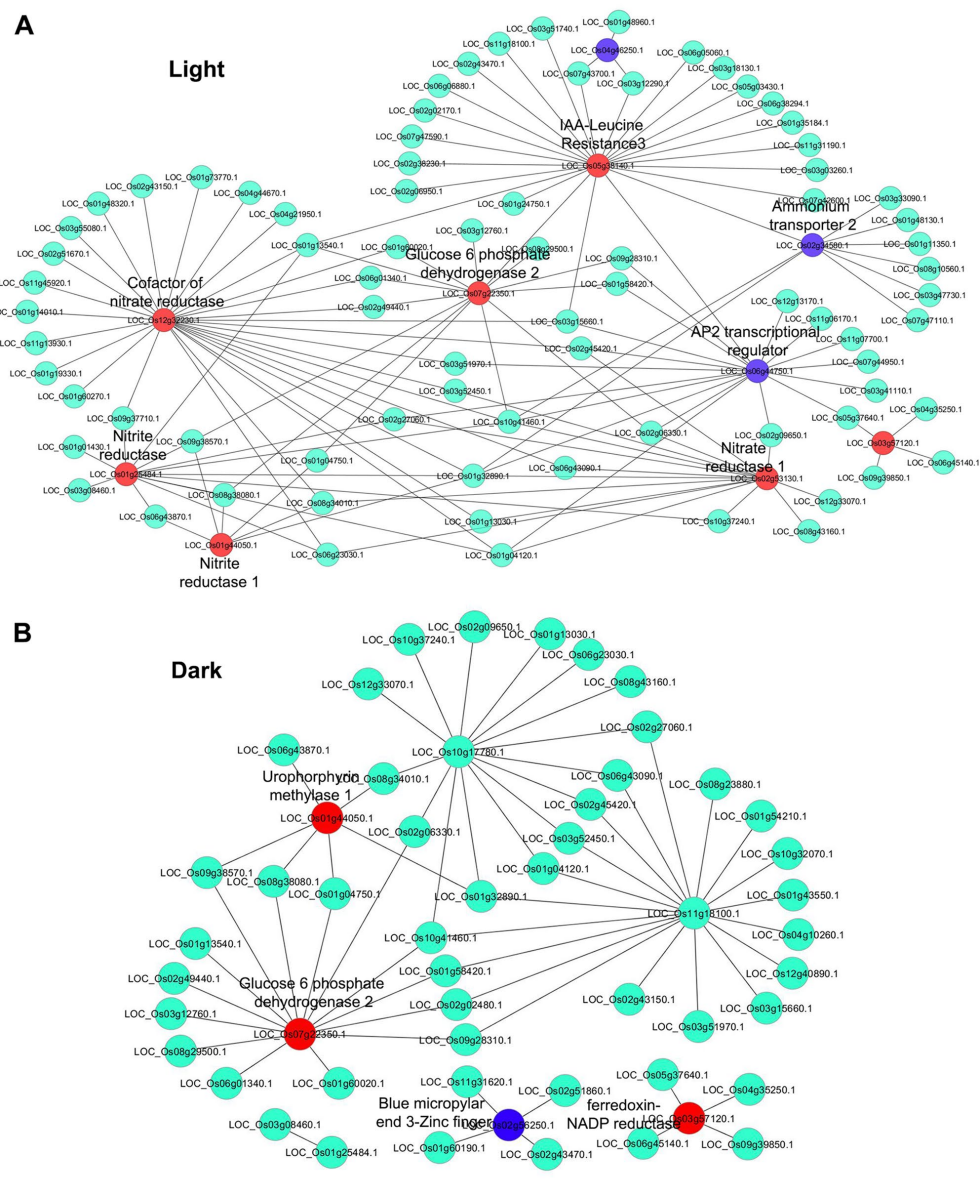
Transcriptional regulatory network of nitrate-responsive genes in light and dark. Nitrate-responsive transcriptional regulatory networks (NTR) in light (**A**) and dark (**B**) were generated using known information in Arabidopsis. NTR networks were constructed in Cytoscape version 3.0.0 (https://cytoscape.org/)^67^ using rice orthologs and the expressions of DEGs were mapped onto the networks. The red and purple nodes represent the up- and down-regulated DEGs and light colour nodes are interactors but not DEGs.

All the nitrate-regulated transcriptional network information was retrieved from published literature^57^ and the corresponding rice orthologs were downloaded from PlantGDB database (https://www.plantgdb.org/). We identified 144 conserved orthologs in rice, with 91 in light and 53 in dark conditions, of which many, but not all were N-responsive in our microarray data (Table S15). We therefore constructed NTR networks associated separately for light and dark conditions in Cytoscape and mapped the expression of the N-responsive DEGs onto the networks (Fig. 8).

We observed well-conserved nitrate-regulated genes such as nitrate transporters, nitrate reductase, nitrite reductase among others in NTR networks (Fig. 8). To check the biological relevance of the constructed NTR networks in rice (Fig. 8), we functionally annotated the NTR networks genes using ExPath GO enrichment tool. The most over-represented GO term for biological processes were regulation of transcription, response to nitrate, nitrate assimilation, biosynthetic process, cellular response to nitrate among others in light, whereasin dark, they were regulation of transcription, nitrate assimilation, cellular nitrogen compound metabolic process, response to water deprivation and auxin-activated signaling pathway among others (Table S16). Interestingly, the GO terms associated with nitrate metabolism were over-represented in light-NTR network as compared to dark-NTR network, confirming that light enhances nitrate-regulation of N-metabolism.

### Nitrate-responsive protein networks reveal overlaps between N-metabolism, development and stress

Nitrate-regulated growth and developmental plasticity of the plant is tightly regulated by light. To understand the underlying interactions at the protein level, we constructed protein–protein interaction (PPI) networks using N-responsive DEGs in light and dark conditions (Figs. S8, S9). The experimentally validated interactors associated with DEGs were retrieved from BioGRID, PRIN MCDRP and STRING databases and networks were constructed and visualized in Cytoscape 3.0.0^66^. We mapped the DEGs expression value onto the networks and nodes were colour coded accordingly. To better understand the PPI networks, sub-clustering of networks were performed using MCODE plugin in Cytoscape, which yielded only one molecular complex from light-nitrate network (Fig. S8) and none in the dark-nitrate network. Light-nitrate associated molecular complex consisted of 3 nodes representing N-metabolic and signaling components viz. NiR, ferredoxin-NADP reductase (FNR) and ferredoxin (Fig. S8), suggesting the potent role of light in nitrate response. To understand the global function of PPI networks for differential nitrate responses in light and dark, the DEG-associated interactors were subjected to GO enrichment analysis using ExPath. It revealed most enriched GO terms for biological processes in light as signal transduction, regulation of transcription, hormone signaling, response to stress and transport among others, whereasin the dark, they were, response to cadmium ion, pentose-phosphate shunt, glucose metabolic process, immune and hormone responses among others (Table S17). This clearly showed that nitrate-regulated signaling differs in the leaves of light-grown and etiolated seedlings.

## Discussion

To date, a number of N-transcriptomic studies were carried out to identify the number of genes and associated pathways involved in N-uptake, -signaling, -metabolism and assimilation among others^17–24^. Light regulation of nitrate assimilation was studied extensively in etiolated and green plants from the point of view of direct light signaling through photoreceptors or indirect signaling through C-metabolites. However, to the best of our knowledge, no study has comprehensively analyzed genome-wide nitrate signaling in light and dark conditions. Therefore, we exploited the availability of functional genomic tools to analyze the nitrate-responsive transcriptome in etiolated and green leaves of rice, a crop notorious for its poor NUE among cereals. This enabled us to understand light-dependent and light-independent nitrate responses, visualize the networks of underlying interactions and identify potential candidates to manipulate N-responses/NUE in rice.

Our experimental design of treating excised leaves by floating them on nitrate solution was aimed at measuring short-term N-response to locally supplied nitrate and to avoid secondary responses^3^, downstream metabolites, or the influence of root-shoot translocation parameters that may respond differently in light and dark^29^. These experimental factors can confound the interpretation of results, whereas we obtained consistent results when cut-leaves were treated with standardized concentrations of nitrate^29^ as was done in the present case. Our transcriptome analyses showed very distinctive sets of nitrate responsive genes in etiolated and green leaves, with only 33 common between them. The common up-regulated genes such as NR, FNR, ENOD20, BTBA4 were involved in N-metabolism while those commonly down-regulated such as peroxidase cysteine-rich repeat secretory protein 55, go35 NBS-LRR were involved in stresses (Table S9). We found opposite regulation of a F-box domain containing protein (OsFBX207), which was up-regulated in light but down-regulated in the dark (Table S9). F-box domain containing proteins are associated with protein degradation and could be one of the major pathways for adaptive mechanism by regulating unfolded protein response (UPR) signaling, specifically through endoplasmic reticulum associated degradation (ERAD) pathway in plants. Our pathway analyses as well as previous transcriptomic study^67^ have shown that many N-responsive genes are associated with degradation pathway in plants. This suggests faster turnover of metabolic processes by light-activated degradation pathways, thus offering new targets to regulate nitrate signaling in the context of damage and repair processes in plants. The stress-associated genes among the down-regulated DEGs common to light and dark conditions suggest crosstalk between nitrate and stress, an underexplored area of immense agronomic importance^26,68^.

GO analyses revealed the nitrate-responsive biological processes that are common or specific to light/dark conditions (Fig. 2, Table S3). N-metabolism, oxidation–reduction and hormone were prominent among the common biological processes regulated by nitrate in both light and dark conditions. However, processes associated with leaf phyllotactic patterning were highly enriched in light (Fig. 2, Table S3). In this context, we found up-regulated expression of APETALA2/ethylene-responsive element binding protein 86 (Os03g0313100) in light. Its up-regulated expression in leaf primordia is known to promote leaf development in Arabidopsis^69^, but its regulation by nitrate/light is a novel finding that links N-nutrition with photomorphogenesis for further investigation, especially in a crop plant like rice. We found the preponderance of biological processes associated with sugar metabolic pathways such as pentose-phosphate pathway (PPP) in dark condition (Table S6). The up-regulation of its rate limiting enzyme glucose-6-phosphate 1-dehydrogenase (Os07g0406300) in the dark in our study may increase the production of NADPH, the reductant for glutamate synthase^70^.

To understand the interactions underlying nitrate signaling and N-response, we compared the N-responsive PPI network in light and dark (Figs. S8, S9). Interestingly, we found that only 39 interactors (~ 8%) were common, while 376 interactors were exclusive to light and 97 exclusive to dark. This could be a reflection of the fact that an equally small % of the DEGs are common to nitrate response in dark and light, indicating very different modes of N-signaling in the two conditions. Importantly, we found significant number of signaling and hormone-associated pathways in the nitrate-responsive PPI networks in light and dark. These findings are consistent with the recent finding that manipulation of N-responsive genes results in the change in hormonal pathways and their crosstalk with those of nitrate^71^. Similarly, both networks revealed DEGs involved in stress response, such as the universal stress protein domain containing protein and MYB family transcription factor, among others. Such crosstalk between nitrate and stress signaling needs further characterization in view of its immense agronomic importance^26,68^.

Sub-clustering of the PPI networks using MCODE did not reveal any molecular complex in the dark, but a single molecular complex was identified in light, consisting of NiR, FNR and ferredoxin (Fig. S8). FNR has been shown to be involved in the regulation of flowering time by modulating the nuclear abundance of blue-light receptor cryptochrome 1 (CRY1) in a nitrate-dependent manner in Arabidopsis^41^. Such a phenomenon is not documented in crop plants and our finding in rice is significant for two reasons: Firstly, it links nitrate and light signaling and is consistent with our other finding that genes related to N-signaling are enriched in light. Secondly and more importantly, flowering time is an important phenotypic trait for NUE in rice (Sharma et al., submitted); its regulation by light offers a crucial meeting point in the mechanism of nitrate response and therefore a potential target to manipulate NUE. Moreover, flowering time is also an important determinant of crop duration in rice and we have already shown that long duration rice genotypes tend to have higher NUE^15^.

An important differentiator of nitrate signaling in light and dark conditions is the differential regulation of various transporters in this study. Major facilitator superfamily (MFS) transporters were among the most abundant DEGs, though their members were different in light and dark conditions (Table S10). They are involved in the transport of nitrate, and sugar among others in plants^72^. Genes involved in the transport of chloride, amino acid, potassium, sulfate, and water channel were also up regulated in light (Table S10). It is known that the accumulation of nitrate in the vacuole is regulated by a chloride channel localized in plant vacuolar membranes^73^. Nitrate and light regulation of all these channels may be important for ionic homeostasis of nitrate and other ions in the cellular pool. This could be a reason for the downregulation of most of the transporters in the dark, such as zinc transporter, ABC transporters, auxin efflux carrier, phospholipid-transporting ATPase. Their downregulation could also be due to less physiological and metabolic activity in dark and the low demand for nutrient uptake/homeostasis.

Another differentiator of nitrate signaling in light and dark is the differential regulation of transcription factors (Fig. 6D and S6). This was explored further using transcriptional regulatory networks we constructed, based on the known information on nitrate-regulatory networks available in Arabidopsis^57^. The constructed NTR networks using rice orthologs were similar to Arabidopsis NTR networks as expected, suggesting a core of nitrate-regulated genes and that their associated connections are highly conserved between rice and Arabidopsis. Mapping the nitrate-responsive gene expression data produced two distinct NTR networks for light and dark (Fig. 8). Their comparison revealed functional connections between nitrate and light signaling, as evident from NR, NiR and AP2 family of transcriptional regulators among others (Fig. 8), which function in a primarily light-dependent manner. Those that function in the dark include FNR1, methylase 1, blue micropylar end-3 zinc finger (BME3-ZF).

We predicted different motifs to be enriched in the promoters of DEGs in dark and light (Tables S12–S14), in view of the previous difficulties in finding nitrate response elements^74,75^. Some of these predicted motifs in N-responsive DEGs up-regulated in light are reported to be bound by well-known nitrate-regulated transcription factors such as TGA1 and GBF1 (Table 1) in Arabidopsis^57,76^. Identification of reliable motifs for nitrate response not only provides a handle for transcriptional manipulation of multiple genes for desired N-response/NUE, but also to construct synthetic promoters for other applications that require an external trigger.

To further characterize interactors of the DEGs identified in PPI and NRT networks, we checked their GO annotations for their biological process as an indicator of their relevance in N-regulated signaling and found that many of them are involved in nitrate signaling in rice. Some of them such as brefeldin A-sensitive Golgi protein-like, DUF581 domain containing protein, inducer of CBF expression 2 are unknown in the context of nitrate signaling/response and are being reported here for the first time as novel candidates for further validation in rice and beyond.

In summary, this study clearly demonstrates the substantial differences in the nitrate-responsive transcriptomes in light and dark conditions as well as in their transcriptional and protein networks. This enabled new insights into nitrate signaling, its cross talk with stress, hormones, developmental pathways and NUE phenotype, revealing potential targets to manipulate N-response/NUE for further validation.

## Methods

### Plant material, growth conditions and nitrate treatments

Seeds of rice *Oryza sativa* ssp. Indica, genotype Panvel1 were obtained from the Kharland Research Station, Maharashtra, India. Seeds were surface-sterilized with 70% ethanol and 0.01% Triton-X 100, washed thoroughly and spread in a plastic tray on washed absorbent cotton presoaked with autoclaved double distilled water. They were grown in a growth chamber for 10 days set at 28 °C, 90 ± 5% relative humidity in total darkness for etiolated plants or under 12/12 photoperiod with 1 kilo lux white light obtained from W/72 Osrambiolux plus fluorescent tubes. The seedlings were watered intermittently. Their primary and secondary leaves were excised and floated on potassium nitrate solution (120 mM) or double distilled water (control) for 90 min. In case of dark experiments, similar treatments were given to excised leaves in dark condition. The control and nitrate-treated leaves were instantly frozen in liquid nitrogen and stored at -80 ºC till use.

### Measurement of mesocotyl and coleoptile length

Ten days old seedlings grown in light and dark conditions were kept in horizontal position and digital images were captured. The lengths of mesocotyl and coleoptile were measured using ImageJ software (https://imagej.nih.gov/ij/download.html). Statistical unpaired T test analyses were performed in the GraphPad Prism 6 software(https://www.graphpad.com/scientific-software/prism/).

## Estimation of chlorophyll content

The chlorophyll content was estimated as described earlier^77^. Approximately 0.5 g of light-grown or etiolated rice leaves were homogenized in 10 ml of aqueous 80% ice-cold acetone and centrifuged at 15,000 g (10,000 rpm) for 10 min at 4 °C. The supernatant was retrieved and 0.5 ml of it was mixed with 4.5 ml of aqueous 80% ice-cold acetone. The absorbance was measured at 663.2 and 646.8 nm and chlorophyll content was calculated using the formula: Ch-a = 12.25 × A663.2—279 × A646.8; Ch-b = 21.5 × A646.8—5.1 × A663.2. Statistical unpaired T test analyses were performed in the GraphPad Prism 6 software (https://www.graphpad.com/scientific-software/prism/).

### Nitrate reductase assay

Nitrate reductase assay was performed after 6 h of treatment with potassium nitrate as described earlier^38^. Briefly, 100 µl of leaf crude extract was added to the reaction mixture containing 5 mM KNO_3_ and 5 mM EDTA in 0.1 M sodium phosphate buffer (pH 7.5) in a total reaction volume of 0.4 ml. The reaction was incubated for 20 min at 25 °C and terminated by adding 0.6 ml of freshly prepared stopping mixture [1:1 ratio of NED (0.1% w/v) and sulfanilamide (1% w/v in 3 N HCl)]. The pink colour was measured spectrophotometrically at 540 nm. NR activity was calculated as nmoles of nitrite/mg protein/min with the help of a standard curve generated using known concentration of nitrite. NR specific activity was expressed as enzyme activity per mg protein and mean data of three independent experiments with internal triplicates were used to plot the graph with standard errors.

### Total RNA extraction and microarray analysis

Total RNA was isolated from leaves frozen after 90 min of treatment with either water (control) or potassium nitrate (120 mM), using modified hot phenol method as described^78^. The RNA pellet was washed with ethanol (70%, 80% and 100%), dried at room temperature, dissolved in DEPC-treated autoclaved water and frozen at − 20 °C till further use. Microarray experiments were performed using RNA isolated from two independent biological replicates as flip-dye replicates at Genotypic Technology Pvt Ltd, Bengaluru, India. The quality and quantity of total RNA were analyzed using Agilent Bioanalyzer as per the manufacturer’s protocol.

Microarray data analyses were performed as described earlier^79^. Labeling was performed using low RNA Input Fluorescent Linear Amplification Kit (P/N: 5184-3523 Agilent, USA).Total RNA was used to synthesize the first and second strand cDNA. Reaction mixture containing 500 ng RNA and 1.2 µl of oligo dT-T7 Promoter Primer in nuclease-free water was incubated at 65 °C for 10 min. Then 4.0 µl of 5 × First strand buffer, 1 µl of 10 mM dNTP mix, 2 µl of 0.1 M DTT, 1 µl of 200 U/µl MMLV-RT, and 0.5 µl of 40U/µl RNaseOUT were added and incubated at 40 °C for 2 h. After CDNA synthesis, 8 µl of NTP mixture, 2.4 µl of 10 mM Cyanine-5-CTP or Cyanine-3-CTP (Perkin-Elmer, USA), 6 µl of 0.1 M DTT, 20 µl of 4 × Transcription buffer, 0.6 µl of inorganic pyrophosphatase, 0.5 µl of RNaseOUT, 0.8 µl of T7 RNA polymerase, and 15.3 µl of nuclease-free water were added to reaction mixture and incubated at 40 °C for 2 h. Qiagen'sRNeasy mini spin columns were used to purify the amplified samples. The cRNA quantity and specific activity were determined using NanoDrop ND-1000 (v 3.2.1.) and samples with specific activity > 8 were used for hybridization. Reaction mixture containing 1,650 ng of each Cyanine labeled cRNA (41.8 µl), 10 × Blocking agent (11 µl) and 25 × Fragmentation buffer (2.2 µl) was incubated at 60 °C for 30 min in dark. The fragmented cRNA were mixed with 2 × Hybridization Buffer (55 µl) and resulting 110 µl mixtures was hybridized at 65 °C for 17 h in an Agilent Microarray Hybridization Chamber with Hybridization Oven. After hybridization, slides were washed with Agilent Gene Expression Wash Buffer I and incubated them for 1 min at room temperature and 37 °C. Slides were washed again with Wash buffer II in similar condition, cleaned and dried by rinsing with acetonitrile and then scanned using Agilent scanner (G2565B) set at 100% laser power. Agilent Feature Extraction software (version 9.1) was used to extract the data, which was normalized as per the recommended Per Spot and Per Chip protocol. This is a 2-color default normalization, *(Per Spot and Per Chip: Intensity dependent (Lowess) normalization)* where each raw intensity value corresponding to the control channel is adjusted using a locally-weighted regression method called Lowess. Each value in the signal channel was divided by the adjusted control value, resulting in the final normalized value. The raw data were analyzed using GeneSpring 9 GX software and submitted at NCBI GEO database (Accession number: GSE12940).

### Functional classification of DEGs and pathway analyses

Gene ontology based functional annotations of the DEGs were performed by Expath 2.0 tool^47^ using default parameters. We have considered only statistically significant (p value < 0.05) GO terms for further analyses. TreeMap (https://www.treemap.com/) software was used to graphically represent the GO enrichments obtained from Expath analysis. Expath 2.0 tool^47^ was also used to perform the comparative pathway enrichment analyses of DEGs in light and dark. Mapping of DEGs onto various biological pathways was done using MapMan version 3.5.1 (https://mapman.gabipd.org/mapman-download)48. The fold change value (log_2_FC) and corresponding p value were used for significant enrichment of DEGs associated pathways in PageMan^80^. Over-represented and under-represented pathways are depicted with red and blue coloured boxes, respectively.

### Subcellular localization of DEGs

To predict the subcellular localization, the amino acid sequences of DEGs were retrieved from RGAP database (https://rice.plantbiology.msu.edu/) and then analyzed by CELLO program (https://cello.life.nctu.edu.tw/) using default parameters for eukaryotes. Subcellular predictions were also made using TargetP 2.0 (https://www.cbs.dtu.dk/services/TargetP/)81.

### qPCR validation of differential gene expression

To confirm the expression pattern of the DEGs, qRT-PCR was performed in three independent biological replicates, with three technical replicates using gene-specific primers (Table S7). All the primers used in this study were designed using online QuantPrime tool (https://quantprime.mpimp-golm.mpg.de/?page=about). DEGs were selected based on their up- and down-regulated expression for described biological pathways. Approximately 2 µg of total RNA isolated from the control and nitrate-treated leaves was reverse transcribed into cDNA (20 μl volume) using cDNA synthesis kit (GCC Biotech, India). Its amplification reaction was carried out in 10 μl volume containing 1 μl of cDNA, 0.5 μl of forward and reverse gene specific primers (10 µM) and 5 μl of KAPA SYBR FAST Master Mix (2X) Universal (Kapa Biosystems, USA). The reactions were performed in Aria Mx real-time PCR system (Agilent, USA). The relative accumulation of transcripts was analyzed by the comparative C(T) method using actin (BGIOSGA013463) as an internal control. Melting curve analyses of the amplicons were used to determine the specificity of qPCR reactions. Statistical unpaired T test analyses were performed using GraphPad Prism 6 software (https://www.graphpad.com/scientific-software/prism/).

### Construction of protein–protein interaction networks and detection of molecular complexes

The lists of experimentally validated interacting proteins for the DEGs analyzed in this study were retrieved from the databases STRING (https://string-db.org/), MCDRP (https://www.genomeindia.org/biocuration/), BioGRID (https://thebiogrid.org/) and PRIN (https://bis.zju.edu.cn/prin/). We mapped the DEGs to the protein–protein interaction (PPI) networks based on experimental score, using Cytoscape version 6.0^66^. To detect the molecular complexes, we used molecular complex detection (MCODE) plugin in Cytoscape. We also downloaded all the Arabidopsis orthologs from PlantGDB database (https://www.plantgdb.org/), which were used to generate and annotate DEGs-associated PPI and nitrate-responsive transcriptional regulatory networks in rice.

### Motif analyses among promoters of nitrate-responsive genes

One kb promoter regions upstream of the translational start site of the DEGs were downloaded from RAPDB database (https://rapdb.dna.affrc.go.jp/tools/dump). The motif discovery oligo analysis tool of Regulatory Sequence Analysis Tools (RSAT) software^82^ was used to identify enriched motifs (6–8 bases oligonucleotides) in the promoter sequences using default parameters. Rice (*Oryza sativa* IRGSP-1.0.42) whole genome was used as the background and motifs were predicted in both the DNA strands.

## Supplementary information

Supplementary information 1

Supplementary information 2

## Data Availability

GEO accession number: GSE12940.
