# Supplementary material for: Transcriptomic and network analyses reveal distinct nitrate responses in light and dark in rice leaves (Oryza sativa Indica var. Panvel1)
Source: Sci Rep. 2020 Jul 22;10:12228. doi: 10.1038/s41598-020-68917-z (PMC7376017; doi:10.1038/s41598-020-68917-z)
Supplement: Supplementary file 1 — Supplementary information 1 [file 41598_2020_68917_MOESM1_ESM.pdf]

Transcriptomic and network analyses reveal distinct nitrate responses in light and dark in rice leaves (*Oryza sativa* Indica var. Panvel1)

Ravi Ramesh Pathak, Annie Prasanna Jangam, Aakansha Malik, Narendra Sharma, Dinesh Kumar Jaiswal, Nandula Raghuram

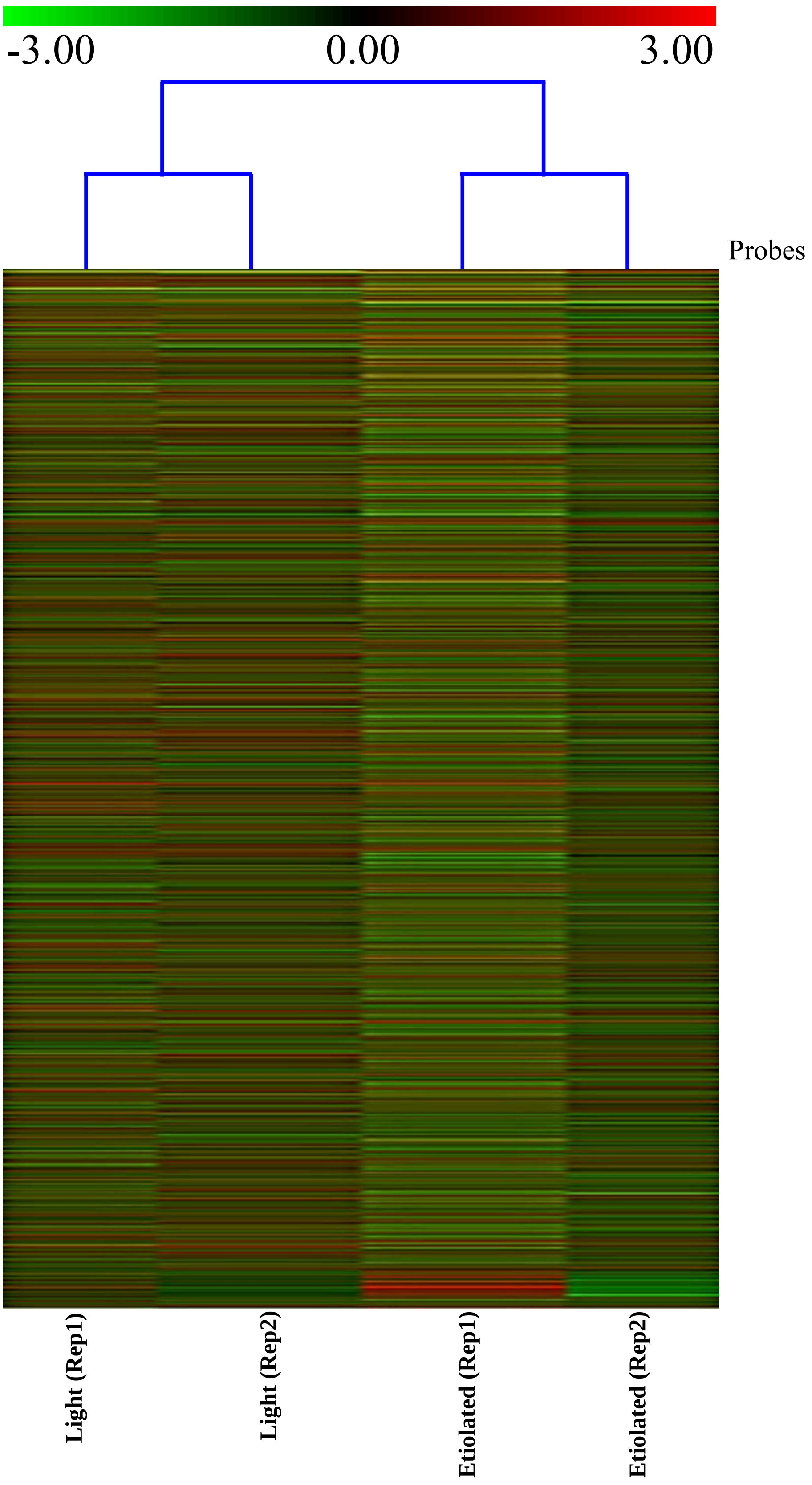

Figure S1. Hierarchical clustering of microarray data in light and dark conditions.

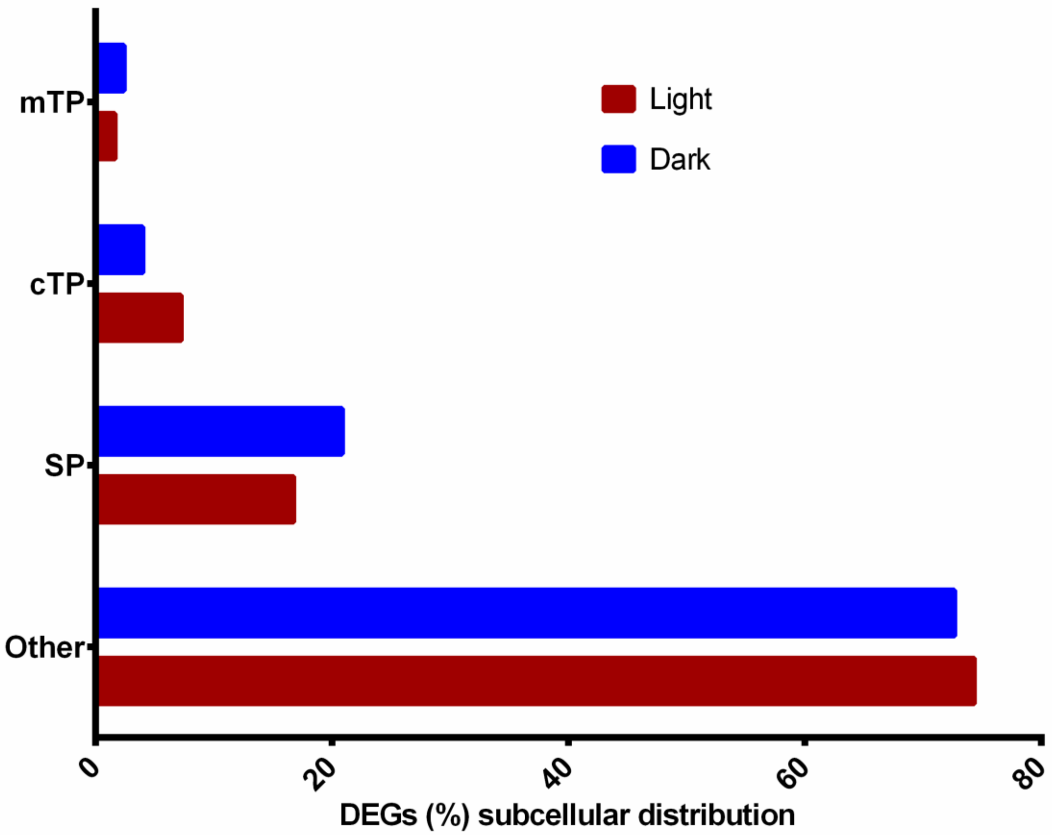

Figure S2. Secretory nature of nitrate-responsive genes encoded protein were predicted using TargetP 2.0. (http://www.cbs.dtu.dk/services/TargetP/). SP: signal peptide; cTPs: chloroplast transit peptides; mTPs: mitochondrial transit peptides.

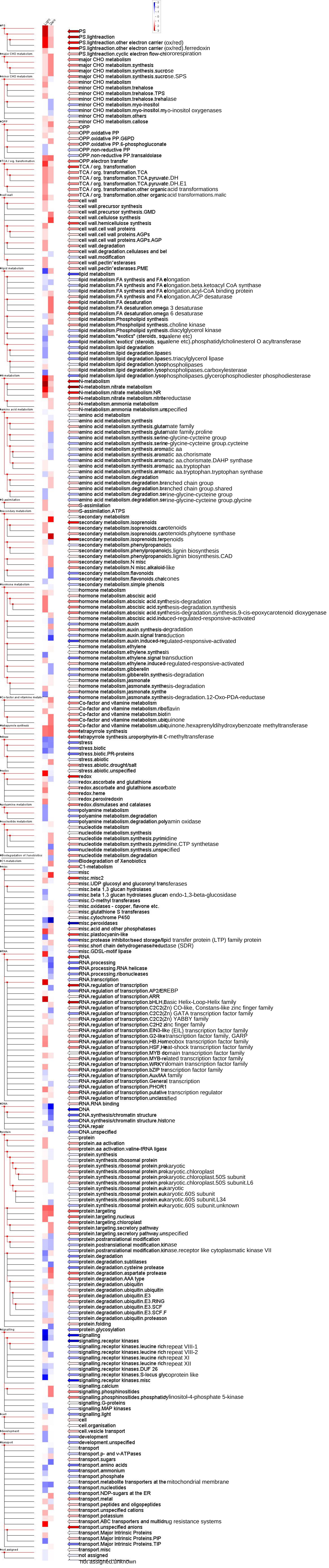

Figure S3. PageMan analyses of nitrate-responsive enriched pathway in light and dark. Wilcoxon test with default parameters was used to predict the enrichment of functional groups. All the significant pathways are represented by arrows based on colour scale (blue, underrepresented; red, overrepresented). For better clarity, pathways name were edited as appropriate in Adobe software (www.adobe.com/).

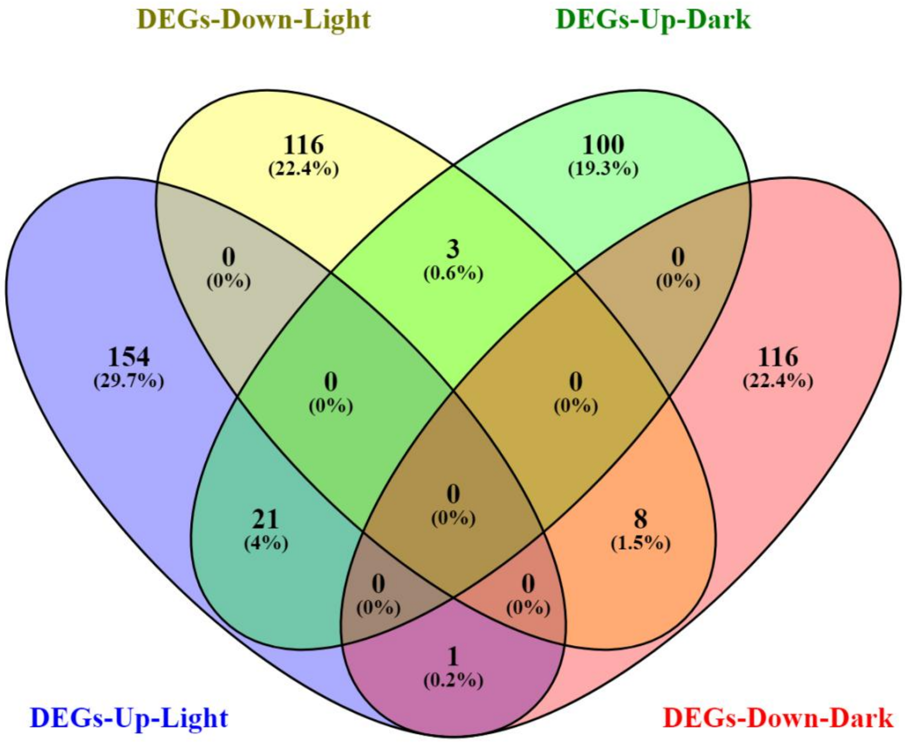

Figure S4. Venn diagram depicting the common and exclusive up-regulated or down-regulated DEGs in light and dark condition. Venn diagram was generated using Venny 2.1.0 (https://bioinfogp.cnb.csic.es/tools/venny/).

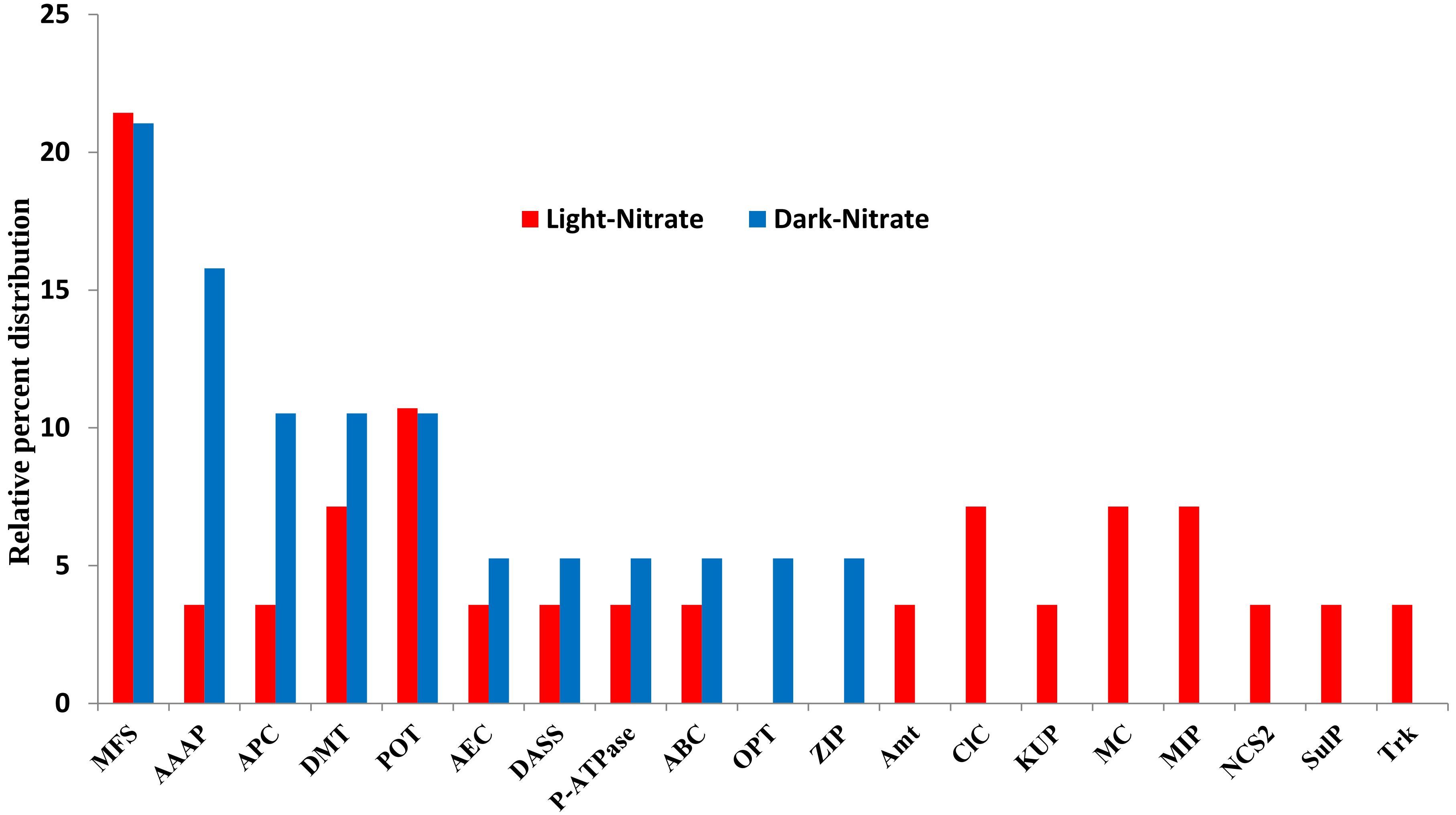

Figure S5. Nitrate-responsive DEGs were searched into transporter database (TransportDB 2.0) and classified them accordingly.
Amino acid/auxin permease (AAAP), ATP-binding cassette (ABC), Auxin efflux carrier (AEC), ammonia transporter channel (Amt), amino acid-polyamine-organocation (APC), chloride carrier/channel (ClC), divalent anion:Na^+ symporter (DASS), drug/metabolite transporter (DMT), K^+ uptake permease (KUP), mitochondrial narrier (MC), major facilitator superfamily (MFS), major intrinsic protein (MIP), nucleobase:cation symporter-2 (NCS2), oligopeptide transporter (OPT), P-type ATPase (P-ATPase), proton-dependent oligopeptide transporter (POT), sulfate permease (SulP), K^+ transporter (Trk), Zinc (Zn^{2+})-iron (Fe^{2+}) permease (ZIP).

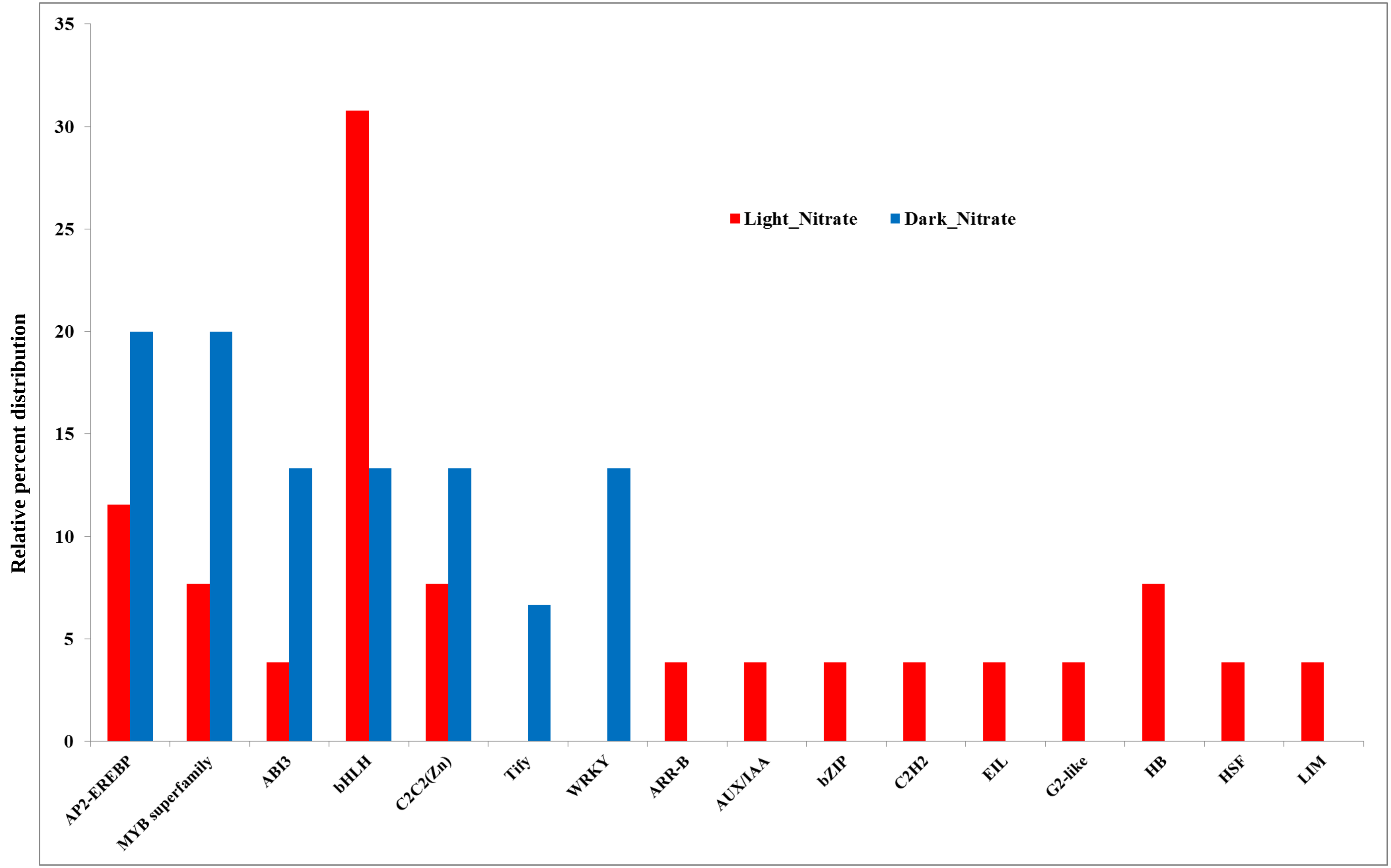

Figure S6. Family-wise classification and distribution of nitrate-responsive transcription factors. Nitrate-responsive transcription factors were mined by searching the DEGs in different transcription factor (TF) databases viz. RiceFREND (http://ricefrend.dna.affrc.go.jp/), RiceSRTFDB (http://www.nipgr.ac.in/RiceSRTFDB.html), STIFDB (http://caps.ncbs.res.in/stifdb/) and MapMan tool. N-responsive TFs were classified into various classes using RiceSRTFDB database.

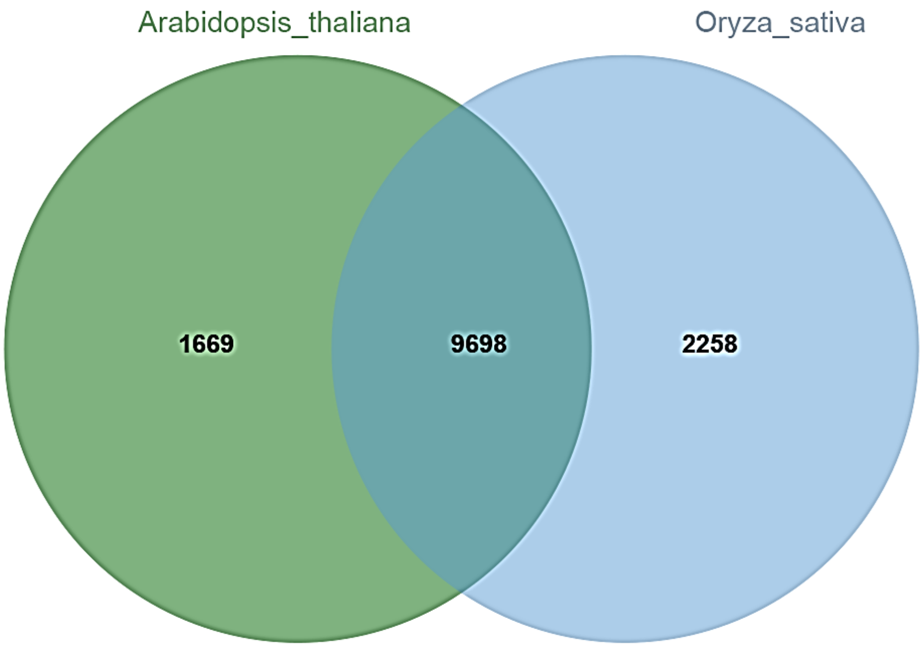

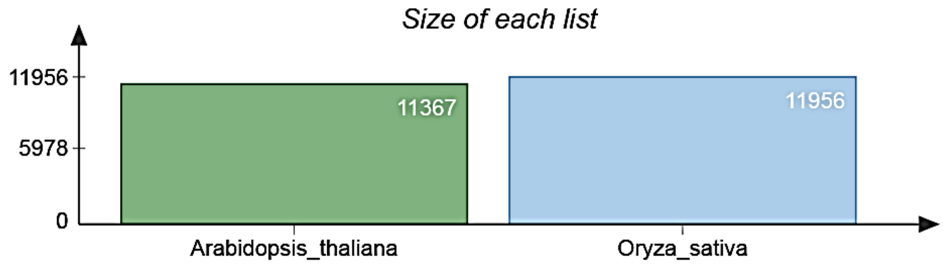

Figure. S7. Venn diagram showing the overlapping orthologous gene clusters between Arabidopsis and rice. This data was generated using OrthoVenn2 tool (https://orthovenn2.bioinfotoolkits.net/home)

A

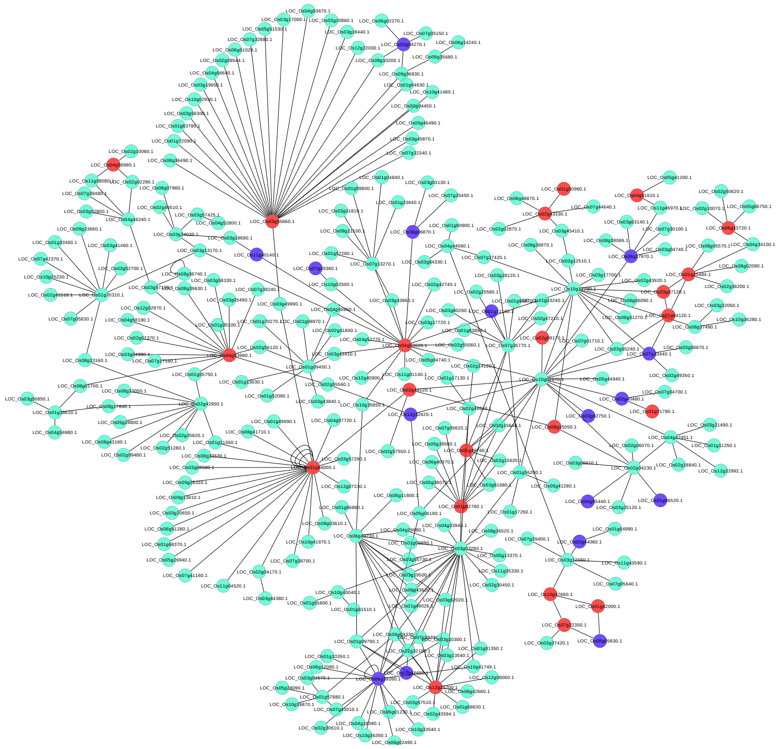

B

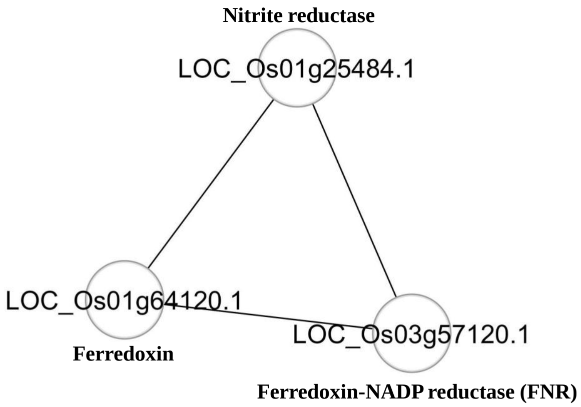

Figure S8. Nitrate-responsive protein-protein interaction (PPI) network in light. (A) All the DEGs identified in light condition were used to retrieve the experimentally validated interactors from STRING, BioGRID, and other databases. The network was constructed in Cytoscape version 3.0.0 (https://cytoscape.org/) and the expressions of the DEGs were mapped into the network. (B) PPI sub-cluster/molecular complex was identified using the MCODE plugin in Cytoscape. The red and purple nodes represent the up-regulated and down-regulated DEGs, respectively. Interactors that are not DEGs are assigned with light sea blue colour.

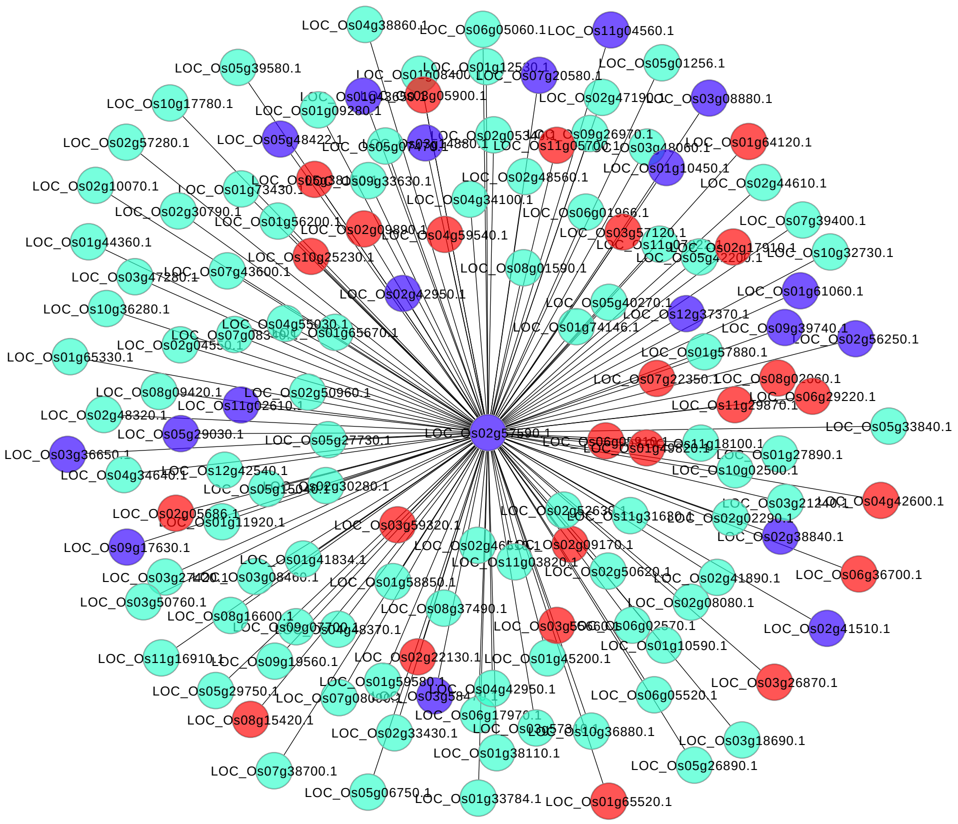

Figure S9. Nitrate-responsive protein-protein interaction network in dark. All the DEGs identified in dark condition were used to retrieve the experimentally validated interactors from STRING, BioGRID,and other databases. The network was constructed in Cytoscape version 3.0.0 (https://cytoscape.org/)and the expressions of the DEGs were mapped into the network. The red and purple nodes represent the up-regulated and down-regulated DEGs, respectively. Interactors that are not DEGs are assigned with light sea blue colour.
